# A Bandwidth-Efficient Dissemination Scheme of Non-Safety Information in Urban VANETs †

**DOI:** 10.3390/s16070988

**Published:** 2016-06-27

**Authors:** Estrella Garcia-Lozano, Celeste Campo, Carlos Garcia-Rubio, Alicia Rodriguez-Carrion

**Affiliations:** Department of Telematic Engineering, University Carlos III of Madrid, Avda. de la Universidad 30, Leganes, Madrid 28911, Spain; emglozan@it.uc3m.es (E.G.-L.); cgr@it.uc3m.es (C.G.-R.); arcarrio@it.uc3m.es (A.R.-C.)

**Keywords:** VANETs, dissemination, urban scenarios, broadcast suppression scheme, store-carry-forward

## Abstract

The recent release of standards for vehicular communications will hasten the development of smart cities in the following years. Many applications for vehicular networks, such as blocked road warnings or advertising, will require multi-hop dissemination of information to all vehicles in a region of interest. However, these networks present special features and difficulties that may require special measures. The dissemination of information may cause broadcast storms. Urban scenarios are especially sensitive to broadcast storms because of the high density of vehicles in downtown areas. They also present numerous crossroads and signal blocking due to buildings, which make dissemination more difficult than in open, almost straight interurban roadways. In this article, we discuss several options to avoid the broadcast storm problem while trying to achieve the maximum coverage of the region of interest. Specifically, we evaluate through simulations different ways to detect and take advantage of intersections and a strategy based on store-carry-forward to overcome short disconnections between groups of vehicles. Our conclusions are varied, and we propose two different solutions, depending on the requirements of the application.

## 1. Introduction

With the aim of avoiding collisions and saving lives, the USA government started a series of actions to standardize and deploy a short-range communications system for vehicles. Other powers, like the European Union (EU) and Japan, have followed and developed their own standards over the last decade. This was the origin of VANETs (vehicular ad hoc networks).

In this type of network, we expect to require the delivery of information (for example, warnings or advertising) to groups of vehicles at more than one hop from the sender. This kind of communication is generally referred to as dissemination of information. In fact, the authors of [1] point out that these two communication patterns will be common in VANETs:

- **Geo-broadcast:** The sender wants to immediately inform all of the vehicles in a larger area about a sudden event. For example, a vehicle that suddenly brakes needs to inform coming vehicles about it. Depending on the message, it may be repeated from time to time and have more relaxed time and delivery requirements, like in the case of work zone warning. The forwarding scheme may be optimized to reduce overhead in cases of high node density.
- **Advanced information dissemination:** The goal is to provide information among vehicles in a given area during a certain time, bridging network partitions. In this case, a wide coverage and the persistence during the intended period are more important than a low latency. Thus, this type of communication usually applies store-carry-forward mechanisms, in which a relay node awaits the appearance of new nodes in its communication range, also called neighbors, before forwarding the message one or more times.

We have focused on creating a dissemination scheme that fits both the relaxed geo-broadcast and the advanced information dissemination patterns: it should minimize the overhead in dense traffic situations (large connected network) and reach as many vehicles as possible in sparse traffic (disconnected small networks). Such a scheme could be useful for public service, improved driving and, especially, business/entertainment applications. As an example, it could be used for disseminating warnings about a blocked road or announcements of scenery overlooks in the route or sales in the downtown area.

According to renowned researchers [2], we must first distinguish between two types of target scenarios, due to their differences in connectivity patterns: roadways and urban areas. In [3], the same authors present a list of challenges that are present in the latter, but not in roadways, that we summarize here:

- **Direction of the message and determination of the region of interest (ROI).** The authors consider that the definition of the ROI depends on the type of application and the vehicles that may be interested in the information. Furthermore, the direction of the message should correspond to the direction of the targets, so defining it may pose a privacy risk.
- **Changes of vehicle direction.** A vehicle may change its current direction at any intersection. This is a problem for choosing nodes for store-carry-forwarding: the selected relay may not go towards the desired direction at some point. The conclusion they reach is that typical mechanisms for roadways are not applicable to this type of scenario.
- **Multiple points to enter or exit the ROI.** As there will be several streets going through the bi-dimensional ROI, vehicles can enter or exit them at different points around the edge. The main implication is that we cannot consider that a dissemination is complete when the message reaches the edge, because it may have covered only a sector of the area of interest.
- **The coverage of a vehicle depends on its location.** The authors point out that vehicles at intersections can connect with vehicles in other streets, while others can only reach vehicles behind or in front of them. They suggest that routing and dissemination protocols should bear in mind this difference in order to take advantage of it.

In addition, they extract two important observations about the special connectivity conditions in urban VANETs from their study. First, the existence of path redundancy is usual: there are multiple ways to connect one vehicle with another. This can help to improve the robustness of routing or dissemination protocols. Second, the broadcast storm and the disconnected network problems are present at the same time. The reason is that vehicles are not evenly distributed in the scenario, forming dense groups disconnected from the others. Urban scenarios are especially sensitive to broadcast storms because of the high density of vehicles in downtown areas. On the other hand, disruptions in propagation caused by buildings and other obstacles accentuate the problem of network partitioning.

Broadcast storms are usually avoided by using broadcast suppression schemes. They consist of a set of rules that prevent some nodes from retransmitting the message. In order to combat network partitioning, a popular type of mechanism is store-carry-forward. One or more vehicles get in charge of keeping a copy of the message and forwarding it after some time (for example, when they encounter other vehicles).

We use both techniques in our search for a dissemination scheme that is useful in urban settings. In this article, we discuss several alternatives that we have considered and present our conclusions about the results achieved. In particular, we develop our work on urban adaptations to the roadway broadcast suppress scheme, which we presented in the original paper. In addition, we design and evaluate different ways to incorporate a specific store-carry-forward mechanism in our solution. We also compare the resulting formula with a protocol from the state of the art, Urban Vehicular Broadcast (UV-CAST). The article is structured as follows: First, in Section 2, we present some of the most relevant work to our approach, as well as our solution for roadway environments. In Section 3, we discuss the design alternatives and the main parameters that affect their performance. Next, in Section 4, we present the metrics that we use, the simulation configuration and the results of the comparative evaluations that we have carried out. Finally, we summarize our conclusions in Section 5.

## 2. Related Work

We divide the contents of this section into two parts. First, we give an overview of the selection of dissemination schemes for urban areas that are relevant to our work. This part concludes with some thoughts about their principal strengths and the mechanisms that they use to achieve them. In the second part, we briefly present our dissemination scheme for roadways, which we use as the base for our version for urban spaces.

### 2.1. Dissemination Schemes for Urban VANETs

Many authors have been interested in the topic of information dissemination in vehicular ad hoc networks (VANETs), and a compilation of relevant works can be found in [4]. There are different techniques that are traditionally used as a base for vehicular multi-hop dissemination. A simple flooding can be used, but it is likely to cause a broadcast storm if the road is heavily populated with vehicles. Most authors use broadcast suppression techniques so that vehicles are able to spread a message through a given area with a lower number of retransmissions. For example, vehicles may construct groupings or clusters a priori with the available information about one-hop neighbors. Then, the pre-selected cluster heads or gateways act as relays when a dissemination occurs. A posteriori techniques usually consist of a contention based on the distance to the previous sender, in order to minimize the number of hops. Others are based on different aspects, entirely probabilistic or a mix of them.

The European Telecommunications Standards Institute (ETSI), who is responsible for developing the Intelligent Transportation Systems (ITS) standards for Europe, has defined GeoNetworkingas the routing protocol intended for ad hoc communications in the 5-GHz band. In particular, we are interested in the GeoBroadcastpacket handling mode; it relies on geo-addressing for a multi-hop dissemination, and it is used for Decentralized Environmental Notification Messages (DENM), i.e., event-triggered updates, among other applications. The forwarding algorithms can be found in the standard specification ETSI EN 302 636-4-1 [5], and they are three: the SimpleGeoBroadcastforwarding algorithm with line forwarding, the Contention-based forwarding (CBF) algorithm for GeoBroadcast, and the AdvancedGeoBroadcast forwarding algorithm. They use the notions of distance-based and location-based contentions, in order to arrive at the destination area in the minimum number of hops and with the idea to avoid the broadcast storm inside it, hence improving the reliability and the speed of the dissemination. They do not apply any special technique that takes into consideration the characteristics that are specific to urban environments.

Given the routing challenges typical of urban areas mentioned above, the research community has identified a necessity to create specific solutions for this type of scenario. Now, we present a selection of works in chronological order that consists of tailored schemes (TAF, ABSM and eMDR), adaptations of general schemes that are better suited for roadways (UV-CAST), and holistic solutions (AMB/UMB).

Urban Multihop Broadcast (UMB) is part of a global solution together with Ad Hoc and Multihop Broadcast (AMB) [6]. It is a MAC level solution that addresses the broadcast storm, hidden nodes and reliability problems in a multi-hop dissemination of messages. It is made up of two phases: directional broadcast and intersection broadcast. Depending on the presence of infrastructure to manage junctions, the intersection broadcast is done by applying AMB (in the absence of it) or UMB (when there is a managing device). UMB is supposed to be used in urban areas because the authors expect that they will be supplied with assisting infrastructure first. The devices located at intersections are responsible for initiating a dissemination branch per every possible direction (except the one that is already covered).

Urban Vehicular Broadcast (UV-CAST) [3] is a version of Distributed Vehicular Broadcast (DV-CAST) [7] adapted to urban environments. It is intended to work in either well-connected or disconnected vehicular networks without help from fixed infrastructure. Depending on the received beacons, a vehicle can determine if it is inside a connected region or not. If it is, it will act according to the well-connected regime, applying a broadcast suppression scheme. Otherwise, be it a boundary node or a totally disconnected node, it will enter the disconnected regime and store-carry-forward the message.

Two Angles Forwarding (TAF), along with two other variations [8], intend to disseminate information in an urban area while meeting the following goals: to be beacon-less, to depend just on the current position and not to make use of any infrastructure. The three algorithms rely on a distance-based contention and on the Triangle Forwarding Rule. This rule is based on the idea that when a vehicle receives the same message from two different neighbors, they form a triangle. By calculating the corresponding angles, a vehicle is able to estimate if it is in a different street than the latest relay. In that case, it becomes a candidate for relay, as well, as it would help the dissemination in a different direction.

In [9], we find Acknowledged Broadcast from Static to Highly Mobile (ABSM), an adaptation of the Parameterless Broadcast in Static to Highly mobile Ad hoc Networks (PBSM) protocol. It is based on the formation of connected dominating sets (CDS) as in PBSM. Vehicles use the beacons from one-hop neighbors to decide whether they belong to a CDS or not. Vehicles at intersections (among others) happen to be in CDSs. These beacons also contain the identifiers of the last received messages, as implied acknowledgments. With the information from beacons and the CDS, every vehicle can construct two lists: neighbors that must have received the message and neighbors that must have not. Then, it forwards the message if the latter is not empty. During the message lifetime, new vehicles that do not acknowledge its reception in their beacons may appear. They are put in the list of ignorant neighbors, and the vehicle will forward again, until the list becomes empty again. This constitutes an implicit store-carry-forward mechanism. This is another totally distributed approach, and the way it solves the problem is similar to UV-CAST.

The goal for the authors of Enhanced Message Dissemination Based on Roadmaps (eMDR) [10] is to reduce the latency. They work on this objective by making vehicles at intersections become relays. This is achieved with the support of GPS and digital roadmaps. A vehicle forwards any new message if its distance to the previous relay is above a threshold or if it is in a different street. This last condition is also true if it is in the same street, but located at an intersection. The vehicle that is closest to the center of the intersection forwards first, inhibiting the rest. The source of the warning emits it periodically, changing the sequence number (that identifies each dissemination). The authors claim that this makes store-carry-forward unnecessary.

A summary of the presented works is in Table 1. There, we can see that the focus is generally set on the efficiency. This is due to the high density of vehicles in cities, together with usually lower speeds. Because of this, most solutions use a distance-based contention or clustering, to select as few relays as possible. There are other works that have their focus on a fast dissemination. These use location-based solutions and binary thresholds in order to take advantage of junctions and to avoid a time-consuming contention. A good portion of them also considers some kind of acknowledgment in order to improve the reliability.

Inside urban areas, buildings are the main problem. The selection of works explained in this section uses different methods for detecting junctions or other streets: digital maps, reception angles and the relative location of neighbors.

There is a discussion regarding disconnected areas that result from buildings blocking the dissemination. Some authors propose using mechanisms to overcome disconnections, while others argue that it should be the source who repeats its message periodically.

Finally, cities are expected to have some infrastructure deployed in them over time. Therefore, dissemination solutions are still capable of working without the aid of any fixed device, but some consider this possibility, as well.

### 2.2. Our Dissemination Scheme for Roadways

We presented in [11] a dissemination scheme that is valid for roadways. Its broadcast suppression algorithm relies on a distance-based contention. Whenever a vehicle hears a new message, it waits a short time, *W*, for an almost simultaneous duplicate of the same message. The distance to the closest vehicle from which it received the same message is stored as $d_{min}$. Then, it assigns itself a forwarding priority based on this distance. This priority is given by the amount of time it waits before forwarding, $t_{w}$, described by Equation (1):

(1) $$t_{w}=T_{max}\times \left(1-d_{min}/r\right)$$

Here, *r* is the communication range and $T_{max}$ is the maximum per-hop delay. The latter is a very important parameter, because it affects the latency of the scheme and it helps to prevent several vehicles from trying to forward almost at the same time. In addition, it has to be small enough to consider the scenario as almost static during the contention. The same value should work properly in very different traffic situations.

This equation lets the vehicle that is furthest from the last relay to forward first. This way, the number of hops (and hence, the number of retransmissions) is minimized.

A vehicle may hear a duplicate of the same message before the wait times out. We assume that messages include a time to live (TTL) field, so any duplicate with a lower value than what the vehicle observed in the first reception indicates that the message is old. Hence, the vehicle aborts the contention. If the duplicate comes in the opposite direction of the first message, that means that another vehicle’s timer expired sooner and got to forward first. In such a case, this vehicle does not have to forward the message. If it comes from a vehicle located over the previous relay, it is ignored because it belongs to the dissemination in the opposite direction. If no other duplicates are received, this vehicle is the one who forwards the message.

In addition, this scheme has a store-carry-forward mechanism to handle isolated groups of vehicles. The scheme does not rely on beacons from other vehicles, and this, in turn, is the cause of its main characteristics: First, the way for a relay to determine if another vehicle has taken its role after its retransmission is to hear a new duplicate with a lower TTL. If it does not hear any new duplicate in a time interval of $W+T_{max}$ after it forwarded the message, it concludes that there is not any new relay, and so, it must store the message and try to find one later on.

## 3. Design of a Scheme for Urban Dissemination

Our starting point is the dissemination scheme that we have just explained in Section 2.2. According to our previous research [11], it achieves a redundancy level close to the optimum in dense roadway environments. However, given the different nature of urban scenarios, we will try to adapt the broadcast suppression scheme in accordance with their singularities in order to obtain good results. The store-carry-forward scheme will also have to respond to the challenges that we summarized in Section 1: the omni-directionality of the message and the difficulty of selecting a suitable relay for carrying, according to [2].

**Figure 1:**
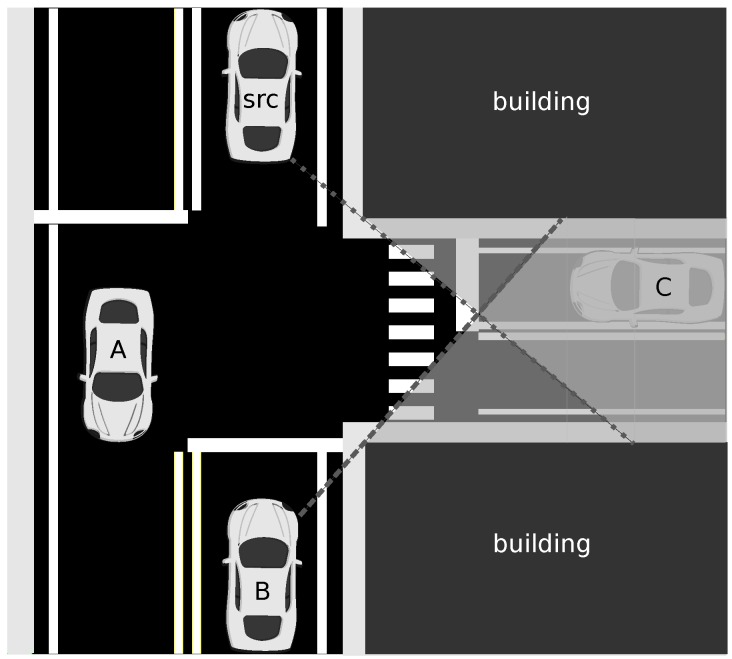
The shadowing problem in urban environments.

We depict a typical situation in a city in Figure 1 to illustrate the case. In this figure, vehicle “src” sends a message that needs to be disseminated. If we apply our roadway scheme, the next relay will be vehicle “B”, because it is the furthest from “src”. However, due to the shadowing effect of buildings, vehicle “C” will not receive the message from “src”, nor from “B”. Vehicle “A” is the only one in the line-of-sight of “C”. Thus, vehicle “A” should be the next relay, so that “C” can also receive the message.

We have broken the problem down into two necessities:

1. an omni-directional and circular approach in the dissemination, because targets may be moving in any direction around the source and
2. an effort to reach neighbors that may be hiding around corners.

We propose to tackle both problems by increasing the chances of forwarding at intersections. Therefore, in order to achieve this, a vehicle that receives a new message first tries to determine if it is at an intersection. We are going to explore two different approaches for this. The first one is using a digital map together with their location coordinates. The other one is interpreting the angle between its direction and the imaginary segment that connects it to the sender. We will describe each one thoroughly below.

Then, vehicles located in an intersection take part in a specific time contention, given by a waiting time called $t_{j}$. The equation that determines its value is related to each of the detection techniques that we are going to use. While vehicles located at intersections use this timer, the others apply the distance-based scheme, waiting $t_{w}$ (see Equation (1)). That is, the intersection contention only affects the vehicles in the same intersection. Conversely, a vehicle located at an intersection will ignore any duplicate coming from outside it.

### 3.1. Broadcast Suppression Mechanism

Basically, the map-based and the angle-based modifications of the base roadway scheme only differ in the method applied to decide if a vehicle or another relay is at a junction.

#### 3.1.1. Map-Based Adaptation for Cities

The first method for detecting junctions that we are considering is the use of a digital map. At the moment of receiving a new packet, a vehicle checks its coordinates to determine if it is at an intersection or not. Given the case, it will use the map again to find out the distance to the junction center, $d_{center}$. It will use this distance to compute a different contention delay, as shown in Equation (2):

(2) $$t_{j}=T_{j}\times \left(d_{center}/r_{junction}\right)$$

Here, $T_{j}$ is the maximum waiting time for vehicles at junctions and $r_{junction}$ is the radius of the given intersection. This radius can be obtained from the digital map, as well. In this case, we want to prioritize the vehicle that is closest to the intersection center, in order to reach as many other vehicles as possible.

We mentioned above that a vehicle located at an intersection will ignore every duplicate, except if it comes from the same intersection. If it is using the map-based method, it will be easy to check the other’s location and its own in the map and see if they belong to the same intersection.

#### 3.1.2. Angle-Based Adaptation for Cities

As a different alternative, we propose that vehicles pay attention to the angle of the previous sender with respect to their own trajectory. We assume that if it is more or less close to a right angle to either side, the message comes from another street. A problem of urban scenarios is that the signal may bounce of and be redirected by buildings, parked cars, street signs, etc. Therefore, learning the reception angle from the antenna may be misleading. To avoid this, we calculate it based on the other’s reported location and the receiver knowledge of its own trajectory. We will use two threshold angles, $\alpha_{min}$ and $\alpha_{max}$, in order to set a range for the condition evaluation. If the reception angle falls between the two of them, the vehicle is considered to be at an intersection. It cannot determine the distance to the intersection center, unlike in the map-based adaptation, so next, it waits a random amount of time, $t_{j}^{\prime }$, from the distribution in Equation (3).

(3) $$t_{j}^{\prime }∼U\left(0,T_{j}\right)$$

Again, the first vehicle to forward inhibits the rest at the intersection to do the same. When using the angle-based method, it will need to use a generic threshold distance, $d_{j}$. If the other vehicle is closer than this distance, we assume that it is located at the same intersection.

### 3.2. Store-Carry-Forward Mechanism

A fact that we must take into account is that we will face frequent disconnections in the vehicular network. The many obstacles that are present in cities artificially break the network, and low traffic densities lead to the same outcome. With the current scheme, the message dissemination stops there.

Depending on the application of the information, this can pose a problem or not. The source may opt to emit the message periodically, and so, vehicles entering the area of interest later on can receive it, as well. In other cases, though, it may be interesting to overcome eventual disconnections. We discuss about how to integrate a store-carry-forward mechanism in our solution for cities in this section.

Being a totally different scenario, we cannot apply our store-carry-forward approach for roadways to an urban scenario. First of all, vehicles’ relative locations are not only behind or in front of another anymore. The message travels omni-directionally, and so do the vehicles. Hence, many of the measures we considered for our scheme for roadways [11] cannot be applied here. However, there are two design principles from the roadway approach that we intend to include in this design, that we proceed to explain.

#### 3.2.1. Acknowledging and Taking over as Relay

When a vehicle forwards a message, it may reach new recipients or none at all. If there is any, it will start a new contention, and there will eventually be a retransmission. Otherwise, no one will forward the message again. Therefore, if disconnections cannot be ignored and we need to apply a healing mechanism, silence after a retransmission will be the sign that it did not reach any new node. A relay that does not hear a subsequent retransmission will have to forward again in the future in the hopes of finding new neighbors. This retransmission will happen at some point in the following interval:

(4) $$\left(W,W+max\left(t_{w},t_{j}\right)\right]=\left(W,W+max\left(T_{max},T_{j}\right)\right]=\left(W,W+T_{max}\right]$$

After the assessment time for receiving almost simultaneous duplicates, *W*, nearby vehicles will start one of two types of contention, depending on the applied algorithm and their position: a regular distance-based contention (given by $t_{w}$ in Equation (1)) or an intersection contention (given by $t_{j}$ in Equation (2) or Equation (3)). Both of them have a maximum length of $T_{max}$ and $T_{j}$, respectively. We want vehicles in intersections to wait, at most, the same as vehicles applying the general distance-based contention equation. Thus, $T_{j}$ must be less than or equal to $T_{max}$. Hence, the maximum time a relay will wait for a new one is $W+T_{max}$.

Conversely, a new duplicate with lower TTL in said interval acts as an implicit acknowledgment of reception and taking over as the relay.

#### 3.2.2. Waiting before Forwarding Again

When we discussed the case of roadways in [11], we adopted **two measures** about how much to wait before forwarding for a second time or more:

1. As we do not use beacons in our scheme, we would not rely on receiving them from vehicles that do not appear in each vehicle’s neighbor database.
2. The best time to forward again is after the vehicle has moved out of the area that it covered with its first retransmission. In order to suit the speed of not only the relay, which may be slow, but also the nearby vehicles, we opted to use the maximum allowed speed, $v_{limit}$. Then, the right time to forward again was every $r/v_{limit}$, being *r* our communication range.

Measure 1 is still applicable to the urban scenario, as our adapted scheme does not relay on a neighbor database. In regard to Measure 2, we are going to try different approaches to finding the best delay between repeated retransmissions from the same vehicle. We have come up with three different alternatives:

##### Speed-Adaptive

A possibility is to modify the equation above in order to adapt the interval to the vehicle’s speed:

(5) $$r/v_{relay}$$

*r* is the vehicle’s communication range and $v_{relay}$, its current speed. The reasoning is that vehicles in cities halt frequently, due to traffic lights, yieldings, pedestrian crossings or jams. It would not make sense to forward at a fixed interval if the vehicles in that spot are not moving or doing so very slowly. If $v_{relay}$ is 0 km/h, it must be checked again after a few seconds in order to compute a finite delay.

##### Fixed Interval

This would be the most similar option to what we included in the mechanism for roadways because the period is fixed. The delay given by Equation (5) might be too long, as the speed limit in urban areas is significantly lower than in roadways. We are going to test a wide range of values, from short intervals (every 0.5 s) up to the long ones that would result in applying the speed-adaptive method at 5 km/h. This range includes the period used in roadways and the corresponding value to the speed limit in urban areas.

##### Map Polling

Finally, we can make the retransmissions happen when the relay is passing through an intersection. This could help the message spread in other directions than the forwarding vehicle’s. Our way to implement this will be via a “map polling”: the vehicle checks its coordinates in the digital map at a fixed interval. If at the time, it is in an intersection, it forwards. Otherwise, it waits for the next check. This will let us test if we can avoid forwarding at every junction (that will surely happen with a low frequency) or not (by using a short period).

The reasoning for the speed-adaptive approach brings the case for a **third measure**: not forwarding if the vehicle has not moved since the last retransmission. If the vehicle has remained stopped, probably the vehicles around it are still the same, and the retransmission would be useless.

## 4. Parameter Tuning and Evaluation through Simulations

In this section, we use realistic simulations for four different goals. First, we perform sweeps on the values for the configuration parameters of the proposed broadcast suppression algorithms. We will use two different city maps so that we can choose the most convenient values for almost any scenario. Second, we will be able to compare the performance of each alternative in its best configuration, in order to choose the final form of our dissemination scheme for urban environments. Third, we want to compare the three different alternatives for store-carry-forwarding and see how much they improve the performance of the scheme. Lastly, we simulate UV-CAST in the same conditions in order to find out how our solution compares to a well-known protocol that has been selected for many other comparisons in the state of the art.

Now, we are going to present the metrics that we will be measuring to compare the results of each algorithm. Next, we detail the configuration details of our simulations, so that they could be replicated. Finally, we present and comment on the results.

### 4.1. Metrics

We can group the metrics we use in this study into three categories: success, overhead and latency.

**Success**: The final goal of a multi-hop broadcast is to reach as many nodes as possible in a given area. We define the success with two different metrics. The *long-reach success* is the proportion of cases where the dissemination goes further than the second hop, i.e., the message leaves the source area. It is normalized to that of simple flooding in the same situation. The other metric is the *coverage*, or the number of vehicles that received the message in the long-reach cases.

**Overhead**: A high overhead is undesirable, as it occupies the limited shared bandwidth with useless data. It can be measured with the *redundancy ratio*, which we define as the ratio of vehicles that act as relays from the total number of receivers. Another metric that echoes the overhead is the *number of lost packets*, as congestion leads to this effect.

**Latency**: The third performance indicator is the latency of the scheme. We may use the per-hop delay or the end-to-end delay. The *per-hop delay* accounts for distance-based and MAC contention and for propagation time. The *end-to-end delay* is averaged over all of the receivers, be they one or several hops away from the source.

### 4.2. Simulations Configuration

As in our preliminary studies [12,13], we have used Veins [14] as our simulation framework because it offers the possibility of importing real maps through OpenStreetMap [15], realistic movements thanks to SUMO [16], an implementation of 802.11p for OMNeT++ [17] and the Simple Obstacle Shadowing [18] propagation model for building shadowing. We have also used VACaMobil [19] in order to maintain a constant traffic density throughout every run of the simulations. The traffic densities go in the range from 25 to 100 vehicles per square kilometer.

**Figure 2:**
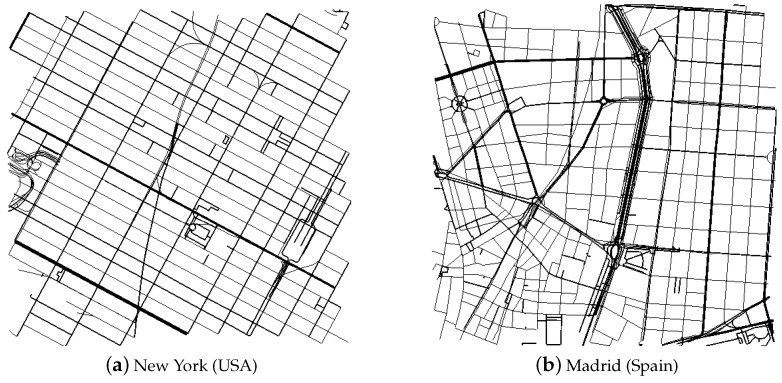
Simulation scenarios for the urban environment.

As discussed in [3,10], the results can be quite different depending on the urban scenario in which the dissemination happens. We have used two different areas of 2 km × 2 km, shown in Figure 2. The Manhattan, New York, map is almost a grid, while the area around Castellana Street in Madrid is more irregular.

After running the simulations, we could appreciate a clear difference in absolute values, which confirms what the mentioned works had already found out: different scenarios yield different results. However, we could also see that the tendency of the curves and the inflection points were always the same for both scenarios. We have already discussed this finding previously in [12]. Therefore, we can extrapolate conclusions from one scenario that will be valid for the rest. Therefore, for the sake of brevity, we only show the results of the New York scenario in this article.

The origin of the message is a fixed unit in the center of the scenario. The preset radius of interest (the ROI) is 1 km. Finally, we have configured the network parameters according to typical values described in [20]. The specific overall configuration is listed in Table 2.

### 4.3. Results

#### 4.3.1. Parameters for Broadcast Suppression

According to the descriptions in Section 3.1, there are three key parameters: $T_{max}$, $T_{j}$ and Δα.

$T_{max}$ is the maximum per-hop delay for a vehicle that is not located at a junction. This parameter is important because it helps to prevent situations in which several vehicles try to forward almost simultaneously. In addition, it has to be small enough to consider the scenario almost static during the contention. The same value should work properly in very different traffic situations. Furthermore, it directly affects the latency of the scheme.

$T_{j}$ is the maximum wait for vehicles at junctions. It must be less than or equal to $T_{max}$, and it affects the performance in a similar way.

Δα is the difference between $\alpha_{max}$ and $\alpha_{min}$. It defines what is considered a junction, given that a vehicle determines its position at one by comparing the reception angle with these two thresholds. As explained above, the reception angle is calculated by using the relay’s reported location and the receiver knowledge of its own trajectory.

From the descriptions above, the reader can note that the base roadway scheme only depends on $T_{max}$; the map-based scheme depends on $T_{max}$ and $T_{j}$; and the angle-based scheme depends on the three parameters. Hence, to isolate their effects, the first step will be to tune $T_{max}$ with simulations of the roadway scheme. Then, we will fix the resulting value for the map-based scheme to find out $T_{j}$. Last, we will configure the angle-based scheme with those values and make a sweep of Δα.

It did not make sense to try to adjust $d_{j}$ by simulations because it is highly dependent on the given city, and we would only overfit it to our scenario. Instead, we have studied the widest diagonal at junctions from a set of four different city maps: New York (USA), Madrid (Spain), Rome (Italy) and Cologne (Germany), available via OpenStreetMap. The results are shown in Table 3. The median, the most usual value, was, respectively, 9.89 m, 9.24 m, 9.64 m and 9.61 m. Accordingly, we have set $d_{j}=10$ m.

**Figure 3:**
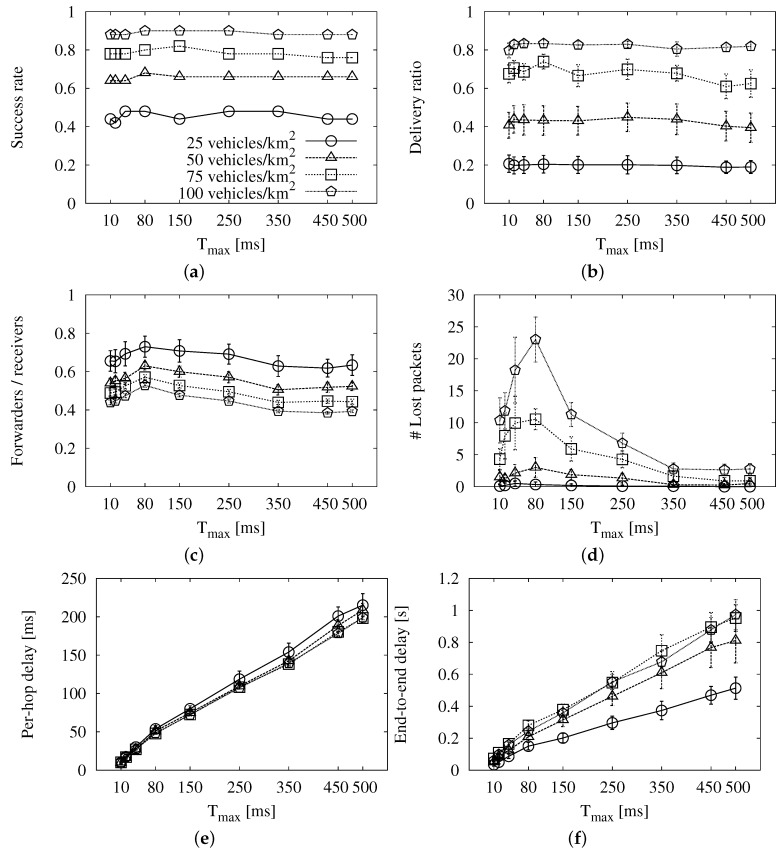
Simulation results of the base roadway scheme with different *T_max_* values in the New York scenario: (**a**) long-reach success rate; (**b**) delivery ratio; (**c**) forwarding ratio; (**d**) number of lost packets; (**e**) average per-hop delay; (**f**) average end-to-end delay.

The results of the simulations to find the best $T_{max}$ using the base roadway scheme are shown in Figure 3. We show the 95% confidence interval together with the values represented in these graphs and the rest that follow.

The first thing we notice is that the long-reach success (Figure 3a) and the coverage (Figure 3b) remain constant for every traffic density considered in our study. Apart from that, we can see that the redundancy ratio in Figure 3c descends when the maximum wait is longer. The reason is that, when the wait is short, there is a high probability that several vehicles will pass the message to the MAC layer almost simultaneously. This leads to the high number of lost packets that we see in Figure 3d for values of $T_{max}$ under 350 ms. Regarding the average per-hop delay (Figure 3e) and the end-to-end delay (Figure 3f), they are proportional to $T_{max}$, as expected. Given that this parameter does not affect the success, we search for a good trade-off between overhead and latency. The former is improved when the value is greater than or equal to 350 ms, while the latency worsens as the value gets higher. Therefore, we choose $T_{max}=350$ ms. This is a rather large value when compared to our chosen value for the roadway scenario, $T_{max}=18$ ms. The reason is that the broadcast storm problem is very pronounced in urban areas, as pointed out in [2]. Apart from the higher traffic density, there are multiple paths that connect the source of the message to any vehicle that is in the same connected network. The access to the channel is more difficult, especially if we face collisions due to several relays forwarding at the same time.

**Figure 4:**
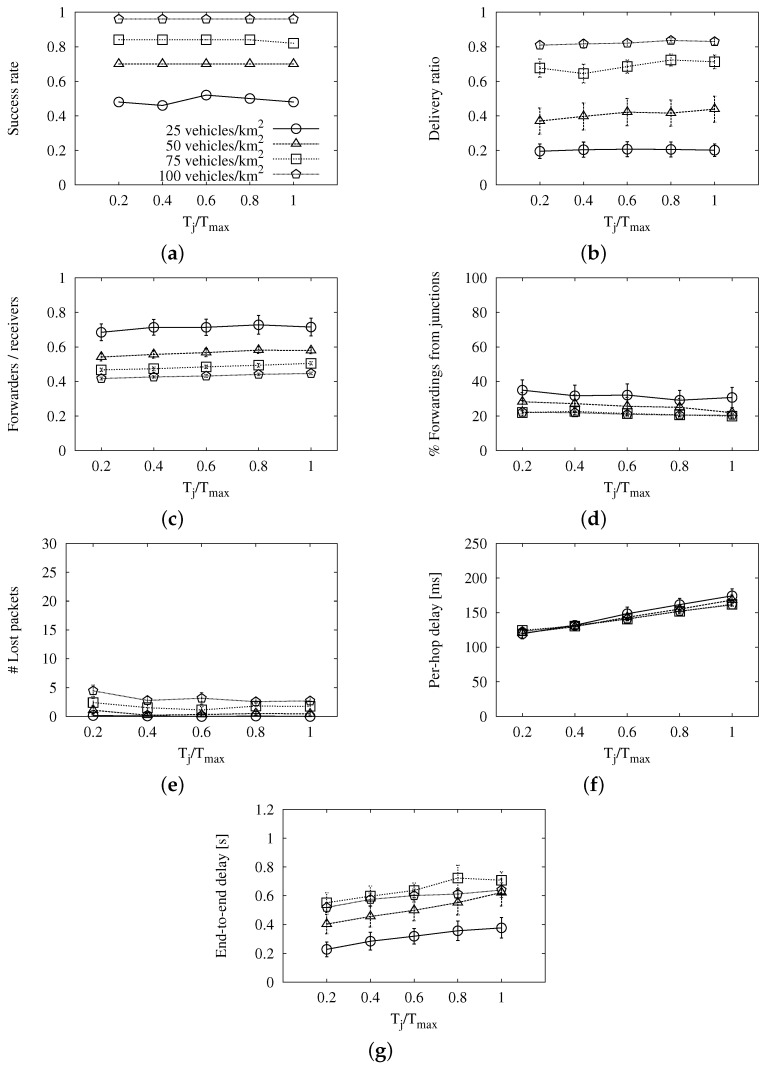
Results of the map-based scheme with fixed *T_max_* and different *T_j_* values in the New York scenario: (**a**) long-reach success rate; (**b**) delivery ratio; (**c**) forwarding ratio; (**d**) portion of relays from junctions; (**e**) number of lost packets; (**f**) average per-hop delay; (**g**) average end-to-end delay.

We use the value we found above, $T_{max}=350$ ms, in the map-based variant to study the effect of $T_{j}$ alone. This parameter is the maximum wait for vehicles at junctions. We want vehicles at intersections to wait, at most, the same as vehicles applying the general distance-based contention equation. Thus, it must be less than or equal to $T_{max}$. In order to better understand the simulated points, the *x*-axis of the graphs in Figure 4 represents the relation between $T_{j}$ and $T_{max}$ instead of absolute values. There, we can see that the success, be it in terms of long reach (Figure 4a) or delivery ratio (Figure 4b), is not related to this waiting time either. We also find a very limited effect in the redundancy ratio from Figure 4c. The slight rise is caused by vehicles at a junction that are also the furthest from the previous relay. They wait so much that another vehicle forwards first, though it does not inhibit them. It is almost balanced out by the slightly higher number of vehicles that forward from junctions (Figure 4d) when their wait is shorter. Again, a very short maximum delay has the same problems of redundancy and losses as $T_{max}$ did, and that we can see in Figure 4e. The average per-hop delay, shown in Figure 4f, is clearly influenced by the vehicles that have to wait less because they are at intersections. However, this effect is hidden in the average end-to-end delay, as we can appreciate in Figure 4g. Following a similar reasoning to that for $T_{max}$, we find that $T_{j}/T_{max}=0.6$ is the best compromise between losses and delay. This corresponds to $T_{j}=210$ ms.

Now, we set to find out the angle thresholds, $\alpha_{min}$ and $\alpha_{max}$. They will let us determine if a vehicle is receiving a message from a different street, by comparing the reception angle with these two thresholds. The reception angle is calculated by using the relay’s reported location and the receiver knowledge of its own trajectory. We define Δα as the difference between $\alpha_{max}$ and $\alpha_{min}$.

**Figure 5:**
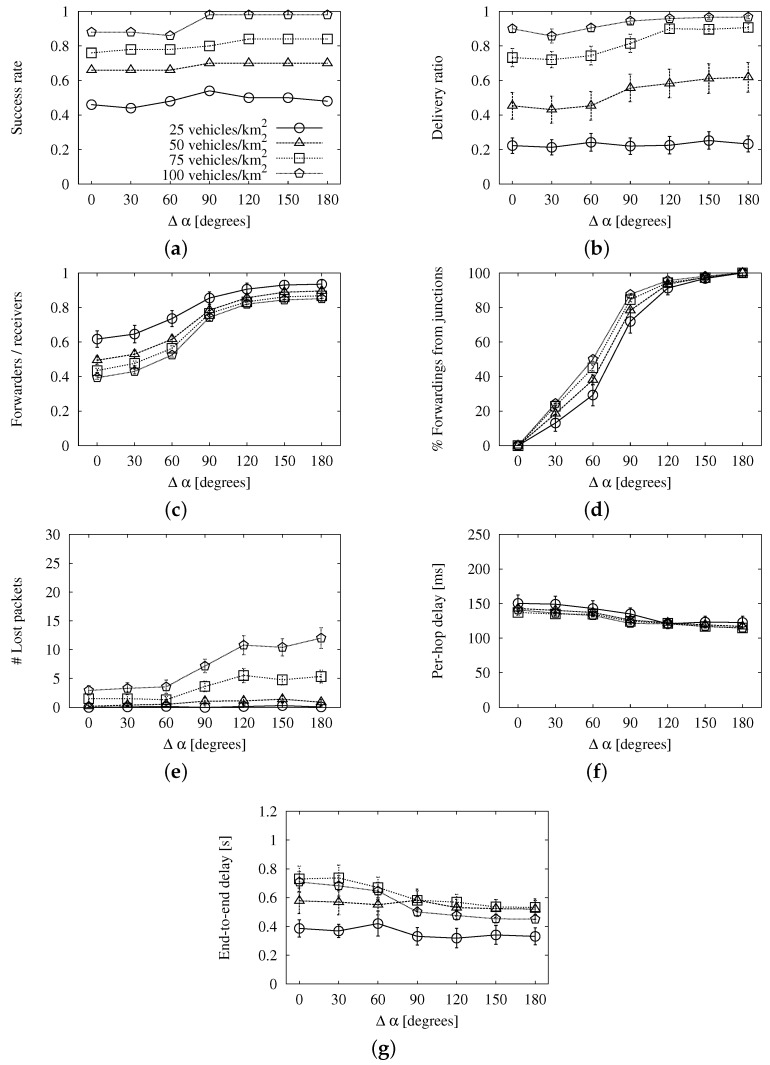
Results of the angle-based scheme with fixed *T_max_* and *T_j_*, and varying Δ*α* in the New York scenario: (**a**) long-reach success rate; (**b**) delivery ratio; (**c**) forwarding ratio; (**d**) portion of relays from junctions; (**e**) number of lost packets; (**f**) average per-hop delay; (**g**) average end-to-end delay.

We can observe the effect of this parameter in Figure 5. When Δα is a flat angle to either side, every reception angle indicates that the vehicle is at an intersection. Hence, all of the retransmissions are assumed to be done from intersections, because every vehicle believes it is at one. Additionally, when Δα is 0 °, no one considers itself inside a junction, so none of the retransmissions is done from one. Δα does not affect the success of the scheme, as we can see in the graphs that represent the long-reach success (Figure 5a) and the delivery ratio (Figure 5b). However, it does have a strong impact on the forwarding ratio, as shown in Figure 5c. When the parameter’s value is a wide angle, many vehicles consider themselves inside a junction. We can see this in Figure 5d. Because of this, the number of retransmissions grows significantly. This leads to many lost packets, as seen in Figure 5e, especially for values bigger than 60. Regarding the latency, we see quite flat curves for the average per-hop (Figure 5f) and end-to-end (Figure 5g) delays. The reduction in latency that we could expect when most vehicles have a shorter waiting time is compensated with the long MAC contentions. All in all, given the high packet loss and latency with wide angles, we limit this parameter to Δα=60.

#### 4.3.2. Comparative Evaluation of the Broadcast Suppression Schemes

In this section, we compare the base roadway scheme and the two urban variants with their chosen configuration. For reference, we have included the simulation results of a simple flooding scheme with a random jitter before forwarding.

The flooding scheme is configured so that when a vehicle receives a new message, it selects a random delay from a uniform distribution. We have selected the maximum value of the delay distribution so that the mean would approximately match the average per-hop delay of the other schemes in the comparison. This way, the dissemination through the whole ROI takes a similar time for all of them. This is important because, as time passes by, vehicles will be entering the ROI (becoming new targets) or leaving it (becoming unreachable). By keeping the end-to-end delays comparable, the number of reachable targets is consistent, as well.

Again, the traffic densities go in the range from 25 to 100 vehicles per square kilometer. The origin of the message is a fixed unit in the center of the scenario, and the preset radius of the ROI is 1 km.

**Figure 6:**
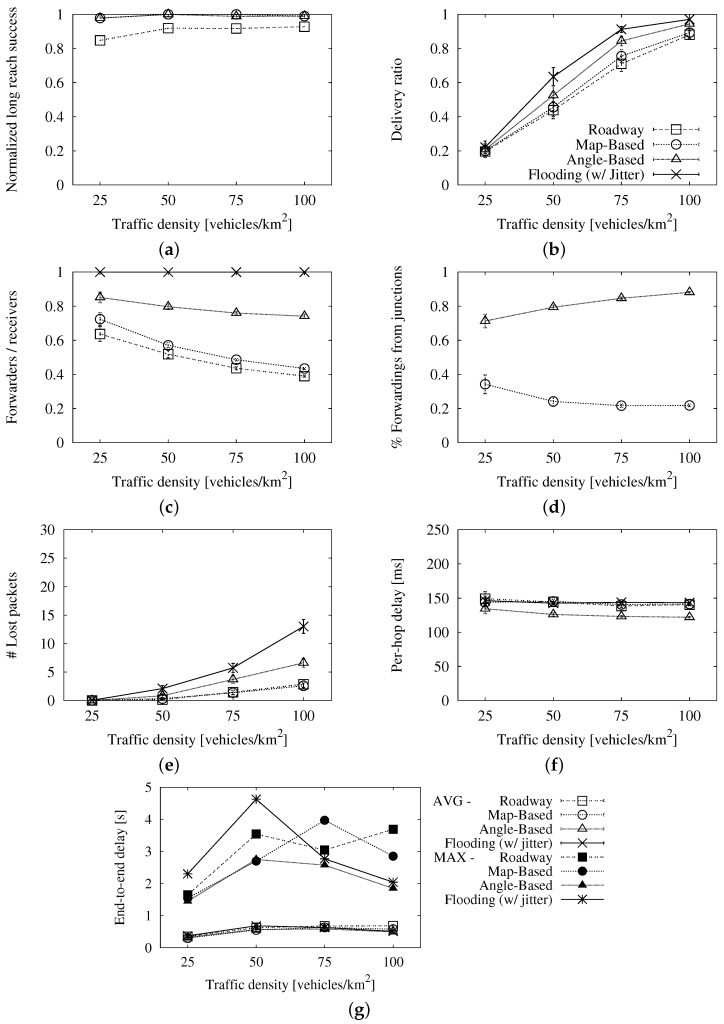
Comparison of the different schemes with the final values in the New York scenario: (**a**) long-reach success rate, normalized to the simple flooding’s rate. (**b**) delivery ratio; (**c**) forwarding ratio; (**d**) portion of relays from junctions; (**e**) number of lost packets; (**f**) average per-hop delay; (**g**) average and maximum end-to-end delay.

The simulation results can be seen in Figure 6. The long-reach success of each one, normalized to that achieved by simple flooding, is shown in Figure 6a. The three studied schemes are close to the flooding, being the two urban adaptations the ones with the highest ratios. We see similar results for the delivery ratio (Figure 6b).

In return for the higher coverage, the urban schemes require more duplicates. This can be noticed in the forwarding ratio in Figure 6c, as well as in the number of lost packets in Figure 6e. As the forwarding ratio tends to one, like in simple flooding, the rising number of duplicates causes a more severe broadcast storm and a higher number of losses. We can see that the angle-based scheme is the worst in this sense, with high ratios of forwarders per receiver even in high densities. We can see in Figure 6d that this is due to the greater number of vehicles forwarding from junctions. An important factor for this is the definition of intersection in each urban scheme. In the angle-based scheme, a vehicle may be physically close to a junction rather than in it, but it will consider itself inside it, whilst the map-based scheme will not allow this. In addition, a map does not account for sidewalks and other open spaces that let the signal travel. Another factor is the distance threshold used in the angle-based scheme to decide if a neighboring relay is in the same intersection as a waiting vehicle. In wide junctions like a big roundabout, it may be too short, letting two or more vehicles in the same intersection forward the message.

Lastly, Figure 6f,g shows the latency of the schemes. Together with the average value, we show the maximum end-to-end delay. Due to the shorter wait for vehicles at intersections, the two urban schemes have a slightly shorter average per-hop delay, though this effect is softened in the average end-to-end delay.

In general, our conclusion is that the adaptations for urban environments improve the success in the dissemination with regard to the roadway scheme, though the latter also offers reasonably good results and with a lower redundancy. Between the two adapted schemes, the angle-based variation achieves a slightly higher coverage than the map-based one at the cost of a relevant overhead. Hence, we think that the map-based option offers a good compromise between gains and losses and that any of the roadway or the map-based schemes would be a good choice for the urban environment.

#### 4.3.3. Comparative Evaluation of the Store-Carry-Forward Strategies

Given that store-carry-forward achieves a higher coverage over time by increasing the number of duplicates, we show the evolution of the performance along the first 450 s since the source emits the message. We use the X axis to represent the time after the emission, and we have chosen four instants to measure each metric: after 1 s, 5 s, 250 s and 450 s. The first milestone corresponds to the first moments of the dissemination, before store-carry-forward can be applied. The next three show the evolution until all of the vehicles that were in the scenario at the beginning of the dissemination have left it.

In this lapse, vehicles keep entering and leaving the scenario (though the density is kept constant thanks to the VACaMobil extension). At each milestone, we need to take into account the activity of the vehicles that have been in the scenario up until that point. The graphs that show the performance results in the following sections reflect this facet.

##### Speed-Adaptive Approach

**Figure 7:**
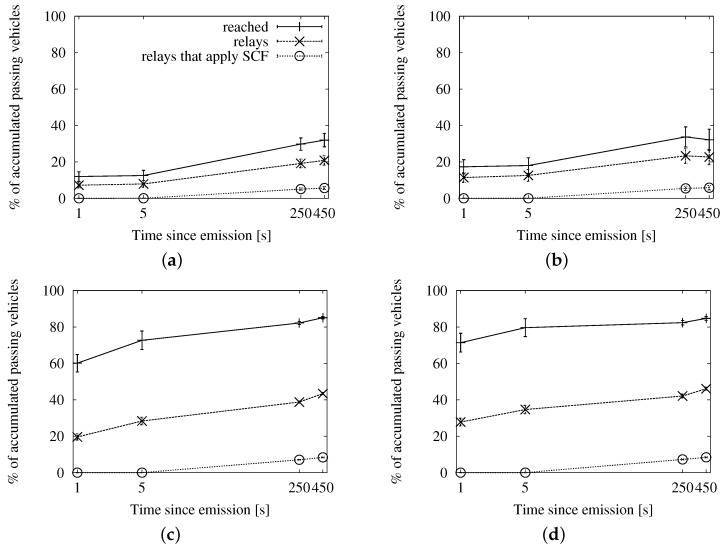
Involved vehicles with the speed-adaptive approach for store-carry-forward in the New York scenario: (**a**) roadway scheme, 25 vehicles/km^2^; (**b**) map-based scheme, 25 vehicles/km^2^; (**c**) roadway scheme, 100 vehicles/km^2^; (**d**) map-based scheme, 100 vehicles/km^2^.

**Figure 8:**
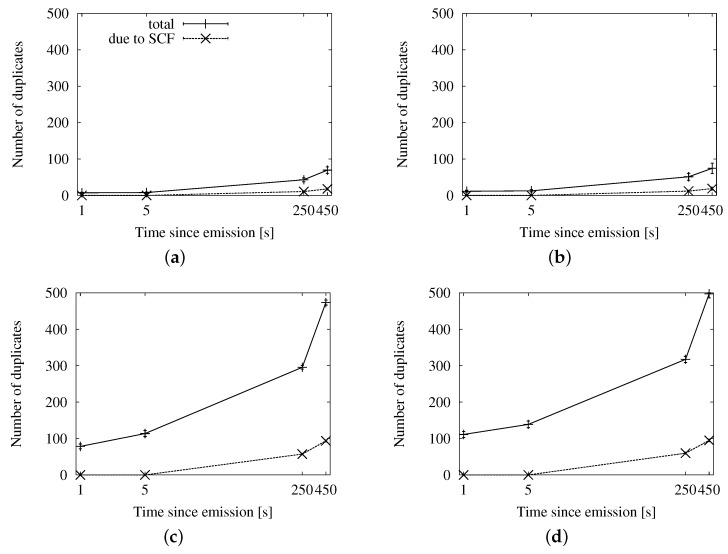
Sent duplicates with the speed-adaptive approach for store-carry-forward in the New York scenario: (**a**) roadway scheme, 25 vehicles/km^2^; (**b**) map-based scheme, 25 vehicles/km^2^; (**c**) roadway scheme, 100 vehicles/km^2^; (**d**) map-based scheme, 100 vehicles/km^2^.

**Figure 9:**
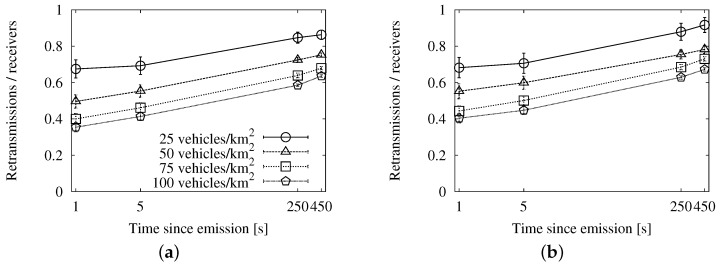
Forwarding ratio or the relation between sent duplicates and receivers with the speed-adaptive approach for store-carry-forward in the New York scenario: (**a**) roadway scheme. (**b**) map-based scheme.

Figure 7, Figure 8 and Figure 9 contain the simulation results when using Equation (5) in Section 3.2.2 to compute the interval between subsequent retransmissions. In this case, we have included the results of applying the store-carry-forward mechanism to the map-based urban scheme, as well. It is worth noticing that the results of the roadway distance-based and map-based adapted schemes are practically the same in the long run. We have cut down on the number of graphs for brevity, but this aspect is also true for the fixed interval and the map polling strategies.

In Figure 7, we can see the delivery ratio along with the portion of vehicles that acted as relays, as well as how many of them applied store-carry-forward. Figure 7a,b correspond to a low traffic density, and thus, they show a higher proportion of relays than what we see in Figure 7c,d. We observe that the portion of relays applying store-carry-forward is 0% until after 5 s since the beginning of the dissemination. The reason is that the speed-adaptive period is larger than that. Given our configured communication range, vehicles should be traveling at more than 167 km/h in order to compute a shorter delay. The absolute portion of relays that apply store-carry-forward after 450 s is slightly higher in the scenarios with 100 vehicles/km^2^. This is, in fact, relatively much lower than in the sparse traffic situations. We could expect this effect, given that large gaps are more prone to occur in the latter.

In Figure 8, we can see the average number of duplicates that were necessary for the dissemination of a single message, together with how many of them were the second or more retransmission from the same relay. As we could already appreciate in the previous set of graphs, the proportion of messages due to store-carry-forward is reduced. The number of messages shows a predictable rise in time as new vehicles enter the ROI and become targets and relays.

Finally, we see the general efficiency at a glance in Figure 9. The ratio of duplicates by receivers goes up slowly, and the number of retransmissions does not reach the accumulated number of targets after 450 s. We will be able to put this information in context when we compare this approach with the other two.

##### Fixed Interval Approach

**Figure 10:**
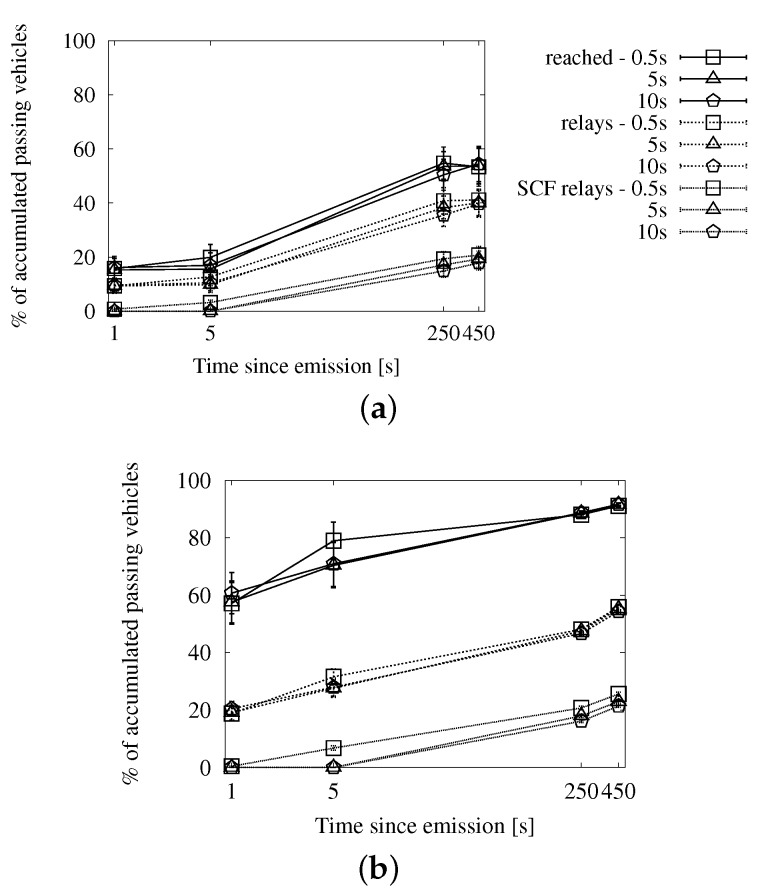
Involved vehicles with the fixed interval approach for store-carry-forward in the New York scenario: (**a**) roadway scheme, 25 vehicles/km^2^; (**b**) roadway scheme, 100 vehicles/km^2^.

**Figure 11:**
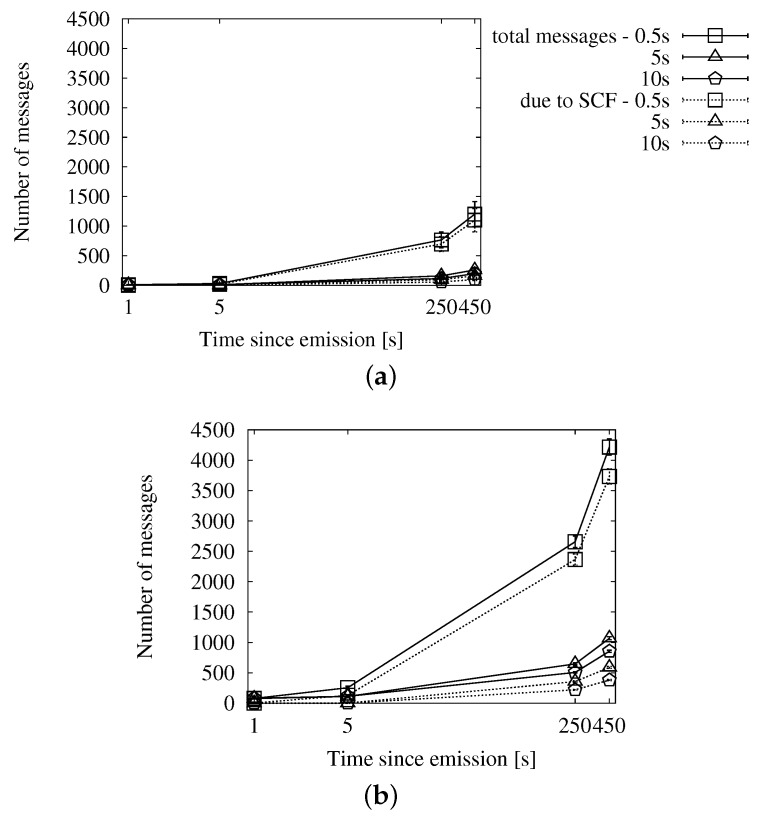
Sent messages with the fixed interval approach for store-carry-forward in the New York scenario: (**a**) roadway scheme, 25 vehicles/km^2^; (**b**) roadway scheme, 100 vehicles/km^2^.

**Figure 12:**
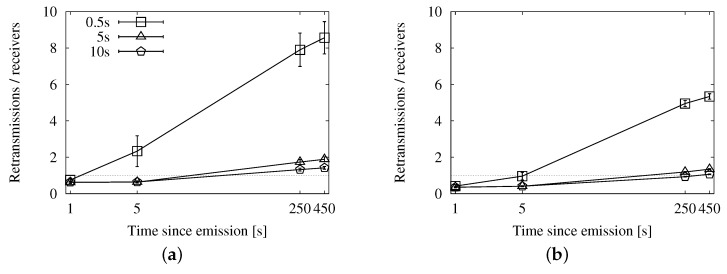
Forwarding ratio or the relation between sent messages and receivers with the fixed interval approach for store-carry-forward in the New York scenario: (**a**) roadway scheme, 25 vehicles/km^2^; (**b**) roadway scheme, 100 vehicles/km^2^.

In Figure 10, Figure 11 and Figure 12, we show the simulation results of applying store-carry-forward to the roadway dissemination scheme with this approach. From all of the tested values, we have selected for representation the three smaller ones (every 0.5, 5 and 10 s).

First, we can see that the aspects we have discussed about the speed-adaptive interval are present in these results, as well. Therefore, we proceed to evaluate how the configured interval length affects the performance.

If we look at Figure 10, we can see that changing the actual configured value does not have a significant impact on the delivery ratio. However, the number of duplicates observed in Figure 11 is clearly higher when the retransmissions due to store-carry-forward are more frequent. All this leads to the higher ratios of retransmissions per reached vehicle that we can see in Figure 10a,b when compared to the other two.

##### Map Polling Approach

**Figure 13:**
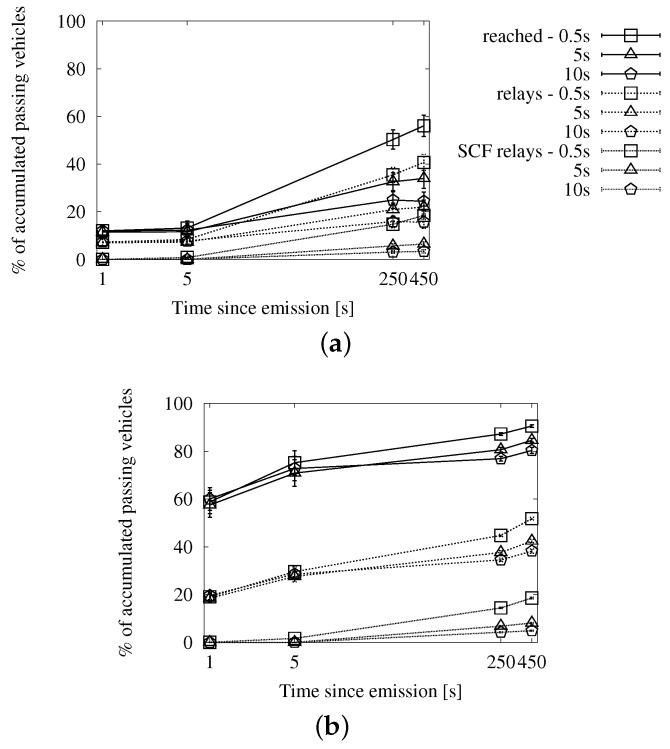
Involved vehicles with the map polling approach for store-carry-forward in the New York scenario: (**a**) roadway scheme, 25 vehicles/km^2^; (**b**) roadway scheme, 100 vehicles/km^2^.

**Figure 14:**
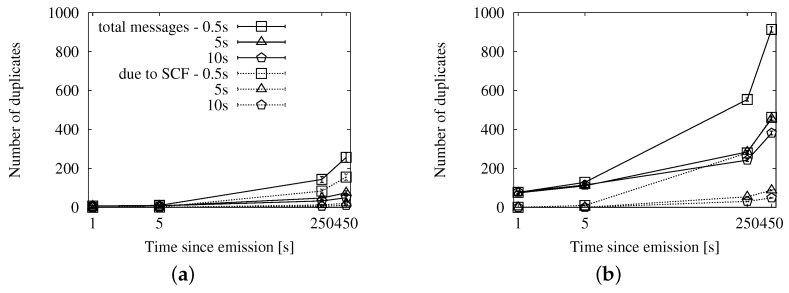
Sent duplicates with the map polling approach for store-carry-forward in the New York scenario: (**a**) roadway scheme, 25 vehicles/km^2^; (**b**) roadway scheme, 100 vehicles/km^2^.

**Figure 15:**
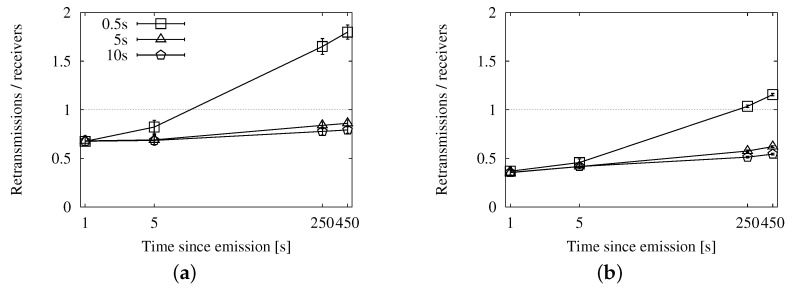
Forwarding ratio or the relation between sent messages and receivers with the map polling approach for store-carry-forward in the New York scenario: (**a**) roadway scheme, 25 vehicles/km^2^; (**b**) roadway scheme, 100 vehicles/km^2^.

We can see the simulation results for this strategy in Figure 13, Figure 14 and Figure 15. The number of reached vehicles, as seen in Figure 13, is slightly lower when the polling period is longer. This is due to the consequent reduction in retransmissions, which we can verify in Figure 14. Additionally, we can see that the highest polling frequency causes a higher proportion of duplicates caused by store-carry-forwarding, up to a half of the duplicates. This contrasts with the results of the other two considered values, which are in the line of the two previous options. This is very clearly seen in Figure 15, where the line for a 0.5-s period rapidly rises above one retransmission per receiver, while the other two remain below during the first 450 s.

##### Comprehensive Comparison

We focus especially on the graphs in Figure 9, Figure 12 and Figure 15, so that we can compare the results from each approach easily. They offer a summary of the performance, as they relate the number of reached vehicles with the total number of duplicates.

First, we have confirmed that, the lower the density, the worse the overhead. Store-carry-forwarding is needed more often, and hence, the number of duplicates increases. However, we have seen that the number of receivers still does not reach the same value as in a higher traffic density.

The speed-adaptive strategy shows a good performance, keeping the ratio under one duplicate per receiver during the 450 s of the experiments. Map polling also offers very good results, as long as the interval is long enough. Checking the map every 5 s yields approximately the same outcome as the speed-adaptive approach. Doing the check every 10 s improves it slightly because the compromise between the number of reached nodes and duplicates is more balanced. The fixed interval strategy, configured with a 10-s retransmission period, also shows very similar results. However, increasing the period for any of these two strategies would not imply better results. The store-carry-forward mechanism would cause less retransmissions at the cost of reaching fewer vehicles.

Regarding the other metrics, the fixed interval approach achieves a higher coverage at the cost of a large number of duplicates, as it almost doubles the speed-adaptive overhead. The map polling option, on the other hand, caused a slightly lower number of messages than the speed-adaptive one, but its coverage was also slightly lower. Therefore, we choose the speed-adaptive strategy, as it is the approach that achieves the best compromise on the different metrics.

#### 4.3.4. Comparison with UV-CAST

We compare the roadway scheme and the map-based urban scheme, together with the store-carry-forward mechanism for cities, with a well-known work from the state of the art, UV-CAST [3]. It is a version of DV-CAST [7] adapted to urban environments. It is intended to work in either well-connected or disconnected vehicular networks without help from fixed infrastructure. From the received beacons, a vehicle can determine if it is inside a connected region or not according to the angles to all of its neighbors. If it is, it will act according to the well-connected regime, applying a broadcast suppression scheme. It consists of a distance-based contention to forward the message first. The waiting time for vehicle *i*, $\tau_{i}$, is calculated using Equation (6):

(6) $$\tau_{i}=\left\{\begin{array}{cc} \frac{1}{2}\left(1-\frac{d_{i,j}}{R}\right)\tau_{max} & \;\text{if}\;i\;\text{is}\;\text{at}\;\text{an}\;\text{intersection}\\ \frac{1}{2}\left(2-\frac{d_{i,j}}{R}\right)\tau_{max} & \;\text{if}\;i\;\text{otherwise} \end{array}\right.$$

where $d_{i,j}$ is the distance from the relay, vehicle *j*, *R* is the maximum transmission range and $\tau_{max}$ is the maximum waiting time. It follows that vehicles that are located at intersections wait half of the maximum wait at most. The authors do not clarify how the vehicle gets knowledge about intersections, but we could assume it gets the information from a digital map. At the end of the wait, if it did not receive any new duplicate, it forwards the message and inhibits the rest. Then, all of the participants become idle again.

If it is a boundary node or a totally disconnected node, it will enter the disconnected regime and store-carry-forward the message. Whenever it receives a new beacon, it forwards the stored message. This is repeated until it exits the area of interest associated with the message.

In the evaluation by the authors, its parameter $\tau_{max}=500$ ms. We modify it to $\tau_{max}=350$ ms, to match our $T_{max}$ value.

**Figure 16:**
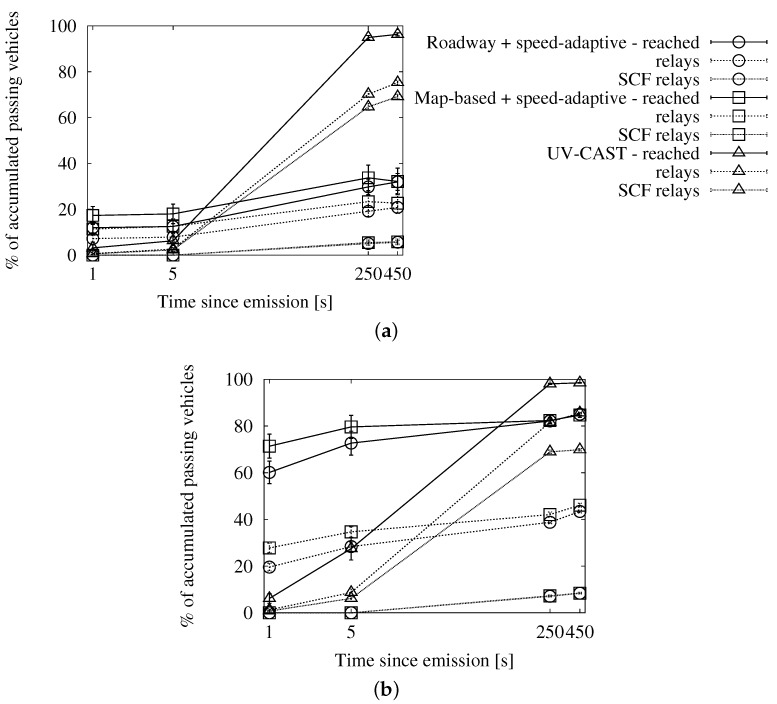
Involved vehicles in comparison with UV-CAST in the New York scenario: (**a**) 25 vehicles/km^2^; (**b**) 100 vehicles/km^2^.

**Figure 17:**
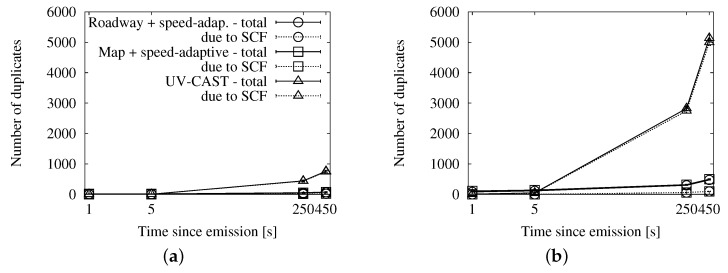
Sent duplicates in comparison with UV-CAST in the New York scenario: (**a**) 25 vehicles/km^2^; (**b**) 100 vehicles/km^2^.

**Figure 18:**
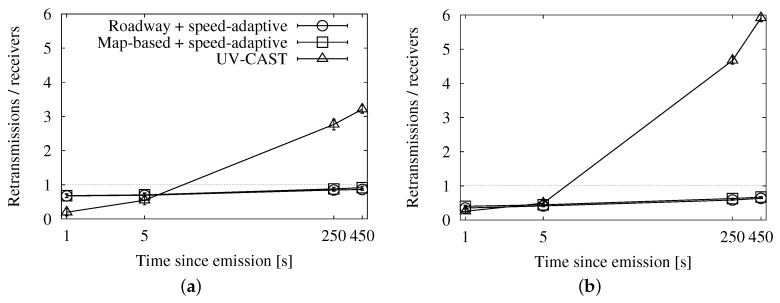
Forwarding ratio or the relation between sent messages and receivers in comparison with UV-CAST in the New York scenario: (**a**) 25 vehicles/km^2^; (**b**) 100 vehicles/km^2^.

The graphs in Figure 16, Figure 17 and Figure 18 show the performance results of our simulations. According to the graphs showing the involved vehicles (Figure 16a,b), we can see that UV-CAST reaches a low number of vehicles in the first stage of the dissemination. However, it achieves an almost total coverage in the long run, even in low traffic densities. Our solutions, on the other hand, get to a higher number of vehicles in the first few seconds, especially in high densities, but the increase over time is limited. The cause of UV-CAST’s high coverage is its overhead, as we can verify in Figure 17a,b. We can see that the vast majority of duplicates when using UV-CAST are due to its store-carry-forward mechanism. This is what lets the protocol reach such a high number of targets.

Finally, the ratio graphs in Figure 18a,b summarize what we have just observed. Our alternatives’ ratios grow together and slowly towards the one duplicate per receiver reference. UV-CAST, for its part, starts with a low ratio, which justifies the low coverage in the early dissemination stage, and rises fast to several duplicates per receiver. It is significant that it causes relatively more overhead as the density is higher, as opposed to our schemes.

## 5. Conclusions

In this article, we have explored how to adapt our distance-based dissemination scheme for roadways to the special characteristics of the urban environment. Our intuition was to add some mechanism that would let vehicles detect when they are passing through an intersection and increase their chance to forward if so. This way, we would increase the chances of the message to be disseminated in other directions and reach more vehicles.

In order to do this, we compared it with two different approaches. One of them is checking the location coordinates in a digital map when a new message arrives. The other is finding out if the reception angle is more or less close to a straight angle or not. Vehicles that estimate that they are in an intersection take part in a different time contention to gain precedence. We have tested the three algorithms thoroughly via simulations.

Our conclusions are varied. First, we have found the most suitable values for the maximum per-hop waiting time, the maximum junction-specific waiting time and the threshold angles. Furthermore, we have discovered that the angle-based approach detects intersections very well and incurs a high overhead due to the intensive forwarding at corners. Finally, we have learned that the adaptation to urban scenarios does not achieve a significantly better performance than the roadway scheme. They do reach more vehicles, but the number of necessary duplicates is also higher.

We still had to try adding a store-carry-forward mechanism for cities, so that we could weigh the extra coverage achieved by the urban scheme. We have created a custom store-carry-forward following the same principles that guided us for the roadway version [11]: We use the reception of a new duplicate with a different TTL as an implicit acknowledgment of reception and taking over as relay. We base the delay before retransmitting for a second time or more on a timer instead of an event. Finally, vehicles must skip forwarding if they are halted.

The timer for retransmissions could be set in different ways, and we have considered three: with a fixed interval, based on the current vehicle’s speed and by polling the position of the vehicle in a digital map to check if it is at an intersection. Again, we have tested the different options via simulations. The performance results we have obtained show that the map-polling and the speed-adaptive approaches are good candidates, though the latter offers a better compromise on coverage and overhead. In addition, we have observed that, no matter the initial coverage of the scheme, in the long run, it gets to the same point in coverage and overhead, depending only on the approach for the retransmissions timer. When compared to UV-CAST, we see that we achieve a better coverage in the first stages with either of the two options, but UV-CAST gets an almost total coverage later, thanks to the high amount of duplicates its store-carry-forward mechanism issues.

Therefore, our final conclusion is that we can apply two different schemes depending on the requirements of the application. If the source will repeat the message periodically, then using store-carry-forward does not make sense, and the map-based urban adaptation should be applied. Otherwise, it is a good idea to use the roadway scheme with the addition of the store-carry-forward for cities.

## Figures and Tables

**Table 1:** Summary of the main works for urban dissemination. UMB, Urban Multihop Broadcast; UV-CAST, Urban Vehicular Broadcast; TAF, Two Angles Forwarding; ABSM, Acknowledged Broadcast from Static to Highly Mobile; CDS, connected dominating sets; eMDR, Enhanced Message Dissemination Based on Roadmaps.

	Goal	Relay Selection	ACKed	Junction Detection	Store-Carry-Forward	Needs Infrastructure	Needs Beacons
UMB, 2007	fast	distance-based	hand-shake	digital map	no	yes	no
UV-CAST, 2011	efficient, resilient to disconnections	distance-based	in beacons	digital map	yes	no	yes
TAF, 2012	efficient	distance-based	no	reception angles	no	no	no
ABSM, 2012	reliable, efficient	clustering (CDS construction)	in beacons	-	yes	no	yes
eMDR, 2012	fast	location-based + distance thresholds	no	digital map	no	no	yes

**Table 2:** Network parameters in the simulations.

Parameter	Value
MAC	802.11p
Frequency band (10 MHz channel)	5.900 GHz
Propagation model	Simple Obstacle Shadowing
Transmission power	1 W
Sensitivity	−77 dBm
SNR	13 dBm
Thermal noise	−104 dBm
LOS communication range (*r*)	232 m
Modulation	QPSK
Bitrate	6 Mbps

**Table 3:** Statistics of the widest diagonal at junctions in several cities.

City	Minimum	Median	Maximum
New York	2.34 m	9.89 m	49.69 m
Madrid	2.56 m	9.24 m	45.09 m
Rome	2.29 m	9.64 m	81.62 m
Cologne	2.31 m	9.61 m	77.80 m
